# Comparison of QuantiFERON-TB Gold Plus results using ELISA and automated CLIA detection: a 39,000-sample real-world study

**DOI:** 10.1128/spectrum.02295-26

**Published:** 2026-10-14

**Authors:** Federica Sorella, Vincenzo Ferraro, Paolo Caiazza, Miriam Ciccarelli, Giuliana Germinario, Alessia Cantiani, Giulia Lombardi, Francesco Bisognin, Paola Dal Monte

**Affiliations:** 1 Department of Medical Science and Surgery, Alma Mater Studiorum - University of Bologna 9296, Bologna, Italy; 2 Microbiology Unit, IRCCS Azienda Ospedaliero-Universitaria di Bologna 9296, Bologna, Italy; Maryland Department of Health, Baltimore, Maryland, USA

**Keywords:** indeterminate results, ELISA, chemiluminescence immunoassay (CLIA), tuberculosis, QuantiFERON-TB Plus, interferon-gamma release assay

## Abstract

QuantiFERON-TB Gold Plus (QFT-Plus) is a widely used interferon-γ release assay (IGRA) for the diagnosis of *Mycobacterium tuberculosis* infection. Recent advances introduced fully automated chemiluminescence immunoassay (CLIA) platforms as alternatives to conventional enzyme-linked immunosorbent assay (ELISA), potentially improving analytical performance and laboratory workflow. This study compared ELISA- and CLIA-based QFT-Plus assays using both lyophilized and liquid conjugate formulations in routine clinical practice. This retrospective study included 39,604 QFT-Plus assays performed during three distinct 1-year periods: 2019 (ELISA), 2023 (CLIA with lyophilized conjugate), and 2024 (CLIA with liquid conjugate). Test results were classified as positive, negative, or indeterminate. Indeterminate results were further categorized as due to low mitogen response (LMR) or high Nil value (HNV). Indeterminate results significantly decreased from 6.5% (777/11,885) with ELISA to 3.1% (394/12,690) with CLIA test using lyophilized conjugate and 3.1% (470/15,029) of CLIA tests using the liquid conjugate (*P* < 0.0001). Positive results accounted for 10.6 (1,254/11,885), 10.3 (1,312/12,690), and 9.0% (1,355/15,029) of tests, respectively. Among indeterminate results, LMR accounted for 97.3% (756/777) with ELISA, whereas HNV accounted for 27.4 (108/394) and 16.8% (79/470) with the lyophilized- and liquid-conjugate CLIA methods, respectively. Agreement between TB1 and TB2 responses was consistently high across all methods (98.5–98.8%; *κ* = 0.92). CLIA-based QFT-Plus assays were significantly associated with a lower proportion of indeterminate results while maintaining an excellent TB1/TB2 concordance compared with ELISA. These findings support fully automated CLIA platforms as a robust and efficient alternative to conventional ELISA for high-throughput tuberculosis screening and routine clinical diagnostics.

**IMPORTANCE:**

This study provides a large real-world evaluation of QuantiFERON-TB Gold Plus testing across different analytical platforms, including more than 39,000 tests performed in routine clinical practice. The transition from conventional enzyme-linked immunosorbent assay (ELISA) to automated chemiluminescence immunoassay (CLIA) was associated with an approximately 50% decrease in indeterminate results from 6.5 to 3.1%, with similarly low rates observed using both lyophilized and liquid conjugate formulations. Beyond documenting this substantial reduction, our study reveals a marked difference in the causes of indeterminate results: low mitogen response predominated with ELISA, whereas high Nil values accounted for a larger proportion of indeterminate results with CLIA. These findings, interpreted together with our previous paired-sample comparison, indicate that differences in quantitative IFN-γ measurement across platforms may affect how samples are classified against fixed decision thresholds.

## INTRODUCTION

In 2024, an estimated 10.7 million people worldwide developed tuberculosis (TB), and 1.23 million died from the disease (1). Interferon-γ release assays (IGRAs) play a critical role in the diagnosis of *Mycobacterium tuberculosis* infection, particularly for the identification of latent tuberculosis infection (LTBI) (2). Although IGRAs cannot distinguish latent infection from active disease, their high specificity and operational advantages make them valuable tools for TB screening, targeted testing of high-risk populations, and broader TB control strategies. QuantiFERON-TB Gold Plus (QFT-Plus) represents the latest generation of IGRA approved by the United States (U.S.) Food and Drug Administration, an evolution of QuantiFERON-TB Gold In-Tube (QFT-GIT) (3). In contrast to QFT-GIT, QFT-Plus was designed to stimulate both CD4+ and CD8+ T cell responses, thereby capturing a broader spectrum of antigen-specific cellular immunity (4). This modification was introduced to improve assay sensitivity, particularly in recently exposed individuals, young children, and immunocompromised patients (3).

Traditionally, the QFT-Plus assay has relied on enzyme-linked immunosorbent assay (ELISA) technology to quantify interferon-gamma (IFN-γ) released following antigen stimulation. Although ELISA is a widely validated and robust technique, it remains only partially automated and requires manual handling, calibration, and trained operators, all of which may limit workflow efficiency in high-throughput laboratory settings.

To address these limitations, chemiluminescence immunoassays (CLIAs) have recently been introduced as alternative IFN-γ detection platforms. CLIAs enable more automated workflows and reduced processing times, making them well suited for routine large-scale laboratory testing (5).

One such advancement is the adaptation of the QFT-Plus assay to the fully automated LIAISON XL analyzer (DiaSorin, Italy), which received FDA approval in 2019. This platform eliminates manual sample handling and batch processing, thereby streamlining assay execution. Preliminary evidence suggests that CLIA-based IFN-γ detection in QFT-Plus provides diagnostic performance comparable to that of the traditional ELISA method (6, 7).

Initially, the LIAISON QFT-Plus assay employed a lyophilized conjugate that required reconstitution before use, introducing potential for analytical variability due to incomplete dissolution. Beginning in 2024, the assay kit was reformulated with a ready-to-use liquid conjugate designed to ensure greater reagent consistency and simplify workflow procedures. Additionally, the activity of the conjugate is influenced by formulation, reconstitution, and storage conditions. We previously performed a head-to-head comparison between ELISA and CLIA with lyophilized conjugate on a small sample size, and we showed that within the linearity range of the test, CLIA yielded higher quantitative IFN-γ values than ELISA, resulting in a higher number of determinate results, and the conversion of samples that were close to the cutoff into positive borderline results (8).

The aim of the present study was to compare QFT-Plus results obtained using conventional ELISA with those generated by the LIAISON QFT-Plus CLIA platform employing either lyophilized or liquid conjugates over three separate 1-year periods (2019, 2023, and 2024). Data collected during the coronavirus disease 2019 (COVID-19) pandemic years (2020–2022) were intentionally excluded to minimize potential bias related to the increased frequency of indeterminate results reported during that period (9).

## RESULTS

The main study findings are summarized in Tables 1 and 2.

**TABLE 1:** QuantiFERON-TB Gold Plus assay performance: comparison across technologies and years^*a*^

	ELISA—2019 *n* (%)	CLIA lyophilized—2023 *n* (%)	CLIA liquid—2024 *n* (%)
Total tests	11,885	12,690	15,029
Positive	1,254 (10.6%)	1,312 (10.3%)	1,355 (9.0%)
Negative	9,854 (82.9%)	10,984 (86.6%)	13,204 (87.9%)
Indeterminate (total)	777 (6.5%)	394 (3.1%)	470 (3.1%)
Indeterminate-LMR	756 (97.3%)	286 (72.6%)	391 (83.2%)
Indeterminate-HNV	21 (2.7%)	108 (27.4%)	79 (16.8%)

^*a*^ LMR, low mitogen rate; HNV, high Nil value. The percentages of LMR and HNV were calculated relative to the total number of indeterminate results.

### Analytical performance of QFT-Plus assays across ELISA and CLIA platforms

In 2019, 11,885 QFT-Plus samples were analyzed using ELISA. Of these, 9,854 (82.9%) were negative; 1,254 (10.6%) were positive; and 777 (6.5%) were indeterminate. Among indeterminate results, 756 (97.3%) were attributable to low mitogen response (LMR), whereas 21 (2.7%) were due to high Nil value (HNV). Among the 11,108 determinate ELISA results, agreement between TB1 and TB2 was 98.5% (*κ* = 0.92). Of the 1,254 positive results, 1,088 (86.7%) showed positivity in both TB1 and TB2 tubes. Among the 67 (5.3%) TB1-only positive samples, median IFN-γ values were 0.46 IU/mL for TB1 and 0.24 IU/mL for TB2. Among the 99 (7.9%) TB2-only positive samples, median IFN-γ values were 0.24 IU/mL for TB1 and 0.45 IU/mL for TB2.

In 2023, 12,690 QFT-Plus samples were analyzed using CLIA with a lyophilized conjugate. Of these, 10,984 (86.6%) were negative; 1,312 (10.3%) were positive; and 394 (3.1%) were indeterminate. Among indeterminate results, 286 (72.6%) were due to LMR and 108 (27.4%) to HNV. Among the 12,296 determinate CLIA results, agreement between TB1 and TB2 was 98.6% (*κ* = 0.92). Of the 1,312 positive results, 1,140 (86.9%) were positive in both TB1 and TB2 tubes. Among the 93 (7.1%) TB1-only positive samples, median IFN-γ values were 0.45 IU/mL for TB1 and 0.22 IU/mL for TB2. Among the 79 (6.0%) TB2-only positive samples, median IFN-γ values were 0.24 IU/mL for TB1 and 0.45 IU/mL for TB2.

**TABLE 2:** Agreement between TB1 and TB2 in determinate QuantiFERON-TB assay results across technologies and years^*a*^

	ELISA (2019)	CLIA lyophilized (2023)	CLIA liquid (2024)
Total determinate results	11,108	12,296	14,559
TB1+/TB2+	1,088 (9.8%)	1,140 (9.3%)	1,182 (8.1%)
TB1−/TB2−	9,854 (88.7%)	10,984 (89.3%)	13,204 (90.7%)
TB1+/TB2−	67 (0.6%)	93 (0.8%)	88 (0.6%)
TB1−/TB2+	99 (0.9%)	79 (0.6%)	85 (0.6%)
Agreement TB1/TB2	98.5%	98.6%	98.8%
*κ* [CI 95%]	*κ* = 0.921 [0.909–0.933]	*κ* = 0.922 [0.911–0.934]	*κ* = 0.925 [0.914–0.936]

^*a*^ *κ* = Cohen’s kappa coefficient.

In 2024, 15,029 QFT-Plus samples were analyzed using CLIA with a liquid conjugate. Of these, 13,204 (87.9%) were negative; 1,355 (9.0%) were positive; and 470 (3.1%) were indeterminate. Among indeterminate results, 391 (83.2%) were due to LMR and 79 (16.8%) to HNV. Among the 14,559 determinate CLIA results, agreement between TB1 and TB2 was 98.8% (*κ* = 0.92). Of the 1,355 positive results, 1,182 (87.2%) were positive in both TB1 and TB2 tubes. Among the 88 (6.5%) TB1-only positive samples, median IFN-γ values were 0.45 IU/mL for TB1 and 0.24 IU/mL for TB2. Among the 85 (6.3%) TB2-only positive samples, median IFN-γ values were 0.25 IU/mL for TB1 and 0.45 IU/mL for TB2.

### Indeterminate result analysis

The distribution of negative, positive, and indeterminate results differed significantly among the three analytical methods (*P* < 0.0001) primarily due to the marked reduction in indeterminate results observed with CLIA compared with ELISA. Specifically, the proportion of indeterminate results was 3.43 percentage points lower with CLIA using the lyophilized conjugate than with ELISA (RD −3.43%, 95% confidence interval [CI] −3.97 to −2.90) and 3.41 percentage points lower with CLIA using the liquid conjugate (RD −3.41%, 95% CI −3.94 to −2.89). In contrast, no significant difference was observed between the two CLIA conjugate formulations.

Analysis of indeterminate result causes demonstrated significant differences in the proportions of LMR and HNV among methods (*P* < 0.0001 for both comparisons). ELISA indeterminate results were almost exclusively attributable to LMR (97.3%), whereas CLIA assays showed a substantially higher proportion of HNV-associated indeterminate results (27.4% for lyophilized conjugate and 16.8% for liquid conjugate).

## DISCUSSION

The QuantiFERON-TB Plus (QFT-Plus) assay plays a key role in the diagnosis of latent and active tuberculosis, particularly in individuals at increased risk of exposure or with uncertain clinical presentations (10). Over time, several methodological variations have been introduced, including conventional ELISA-based assays and fully automated chemiluminescent immunoassays (CLIAs) employing either lyophilized or liquid conjugates. These platforms differ in reagent formulation, calibration strategies, and detection systems, all of which may influence analytical performance and result interpretation (7).

In this large retrospective study including more than 39,000 QFT-Plus assays, the transition from ELISA to CLIA-based platforms was associated with a substantial improvement in test determinacy. The proportion of indeterminate results decreased from 6.5% with ELISA to 3.1% with both CLIA formulations, representing an approximate 50% reduction.

A major finding of this study was the change in the pattern of indeterminate results following the introduction of CLIA-based testing. We excluded data from the COVID-19 pandemic years (2020–2022) to reduce potential bias from the higher frequency of indeterminate results (9).

Most indeterminate ELISA results were attributable to low mitogen response (LMR) (97%), whereas CLIA assays showed a markedly higher proportion of high Nil value indeterminate results (HNV) (17–27%). These findings suggest that elevated background IFN-γ levels may be influenced, at least in part, by analytical factors related to assay formulation and detection technology rather than exclusively reflecting biological variability.

Our previous head-to-head comparison of 419 identical QFT-Plus specimens analyzed by both ELISA and CLIA demonstrated that, within the assay linearity range, CLIA yields systematically higher quantitative IFN-γ values than ELISA. This difference was particularly relevant around fixed decision thresholds. In that paired study, specimens that were indeterminate by ELISA because of LMR but became determinate by CLIA showed an increase in median Nil-corrected Mitogen from 0.34 to 0.94 IU/mL. Similarly, ELISA-negative/CLIA-positive specimens had median TB1 and TB2 values close to the positivity threshold by ELISA (0.21 and 0.24 IU/mL, respectively) but higher values by CLIA (0.49 and 0.51 IU/mL, respectively) (8).

These previous paired observations provide a plausible analytical framework for interpreting the different distribution of indeterminate results observed in the present large real-world study. Because LMR and HNV are defined using fixed quantitative thresholds (Nil-corrected mitogen < 0.5 IU/mL and Nil > 8.0 IU/mL, respectively), differences in quantitative signal distribution between analytical platforms may alter the proportion of specimens falling on either side of these thresholds. Accordingly, the lower frequency of LMR and the higher relative frequency of HNV observed during the CLIA periods should not necessarily be interpreted as evidence of superior diagnostic performance, but may partly reflect differences in quantitative signal measurement relative to fixed decision thresholds.

However, unlike our previous paired study, the present analysis compares specimens tested during different calendar periods. In fact, a limit of this study is that analytical effects cannot be separated from potential temporal changes in the characteristics of the tested populations (e.g., changes in patient demographics, indications for testing, prevalence of tuberculosis infection, inpatient/outpatient composition, immune status, immunosuppressive treatment, or underlying disease), and both factors may have contributed to the observed differences. Because clinical and demographic variables were not available in the present data set, these potential confounding effects could not be quantified. Furthermore, positivity decreased from 10.6% during the ELISA period to 9.0% during the liquid-conjugate CLIA period. In the absence of clinical, demographic, and testing-indication data, this change may reflect differences in case mix, analytical effects, or both.

Agreement between TB1 and TB2 antigen tubes remained consistently high across all analytical platforms, with *κ* values indicating excellent concordance. These findings support the robustness of the dual-antigen design of the QFT-Plus assay and suggest that inclusion of the TB2 tube enhances assay reliability without introducing substantial analytical variability. The stability of TB1/TB2 concordance across different platforms further indicates that methodological differences primarily affect quantitative signal intensity rather than antigen-specific immune recognition (11).

In addition to analytical performance, CLIA platforms offer several important operational advantages over conventional ELISA, including full automation, reduced hands-on time, elimination of batch processing, and lower reagent consumption. These characteristics improve laboratory efficiency and standardization, particularly in high-throughput diagnostic settings (5, 6).

In conclusion, in this large retrospective study, QFT-Plus testing performed during the CLIA periods was associated with a lower proportion of indeterminate results than testing performed during the ELISA period, while TB1/TB2 concordance remained high across methods. Because the analytical methods were applied during different calendar periods to non-paired populations, and no independent reference standard or clinical information was available, these findings should not be interpreted as evidence of superior diagnostic accuracy. Prospective paired-sample studies are required to determine the extent to which the observed differences reflect analytical characteristics of the platforms, conjugate formulation, or differences in the populations tested.

## MATERIALS AND METHODS

### Study design and samples

This is a retrospective observational study. Eligible tests included all QFT-Plus assays performed as part of routine diagnostic activity during the predefined study periods (January–December 2019, January–December 2023, and January–December 2024) for which a valid final QFT-Plus classification was available. The years 2020–2022 were excluded *a priori* because of the potential influence of the COVID-19 pandemic on the frequency of indeterminate QFT-Plus results.

Blood samples were collected into QFT-Plus blood collection tubes (Nil, TB1, TB2, and mitogen) and incubated at 37°C for 16–24 h within 16 h of collection. Following incubation, samples were centrifuged at 2,700 × *g* for 15 min at room temperature, and plasma was collected for interferon-gamma (IFN-γ) quantification. IFN-γ concentrations were measured using two analytical platforms according to the study period:

1. ELISA-based detection (in 2019) was performed using the SkyLab automated system (Dasit, Italy).
2. CLIA-based detection was performed using the LIAISON XL analyzer (DiaSorin, Italy), employing either a lyophilized (in 2023) or liquid (in 2024) conjugate.

All assays were performed according to the manufacturers’ instructions and recommended protocols (12, 13).

Results were interpreted according to the manufacturer’s criteria and classified as positive, negative, or indeterminate.

A positive result was defined as a TB1 and/or TB2 antigen response ≥ 0.35 IU IFN-γ/mL after subtraction of the Nil tube value. A negative result was defined as TB1 and TB2 responses below this cut-off.

Indeterminate results were defined according to two criteria:

(i) Low mitogen response (LMR): mitogen IFN-γ value < 0.5 IU/mL, indicating insufficient immune responsiveness.

(ii) High Nil value (HNV): Nil tube IFN-γ value > 8.0 IU/mL, indicating elevated background IFN-γ levels.

Indeterminate QFT-Plus results were not systematically repeated as part of the analytical protocol used for this retrospective study. The analysis was based on the final results recorded in the laboratory information system during routine diagnostic activity

Clinical data were not collected for this study. Informed consent was not required, as the data were analyzed anonymously. The study was conducted in accordance with the Declaration of Helsinki.

The study protocol (code 616/2021/Oss/AOUBo) was approved on 21 July 2021 by the Independent Ethics Committee of Area Vasta Emilia Centro (CE-AVEC) of Emilia-Romagna Region, Italy.

### Statistical analysis

Comparisons of categorical variables among years and testing methods (ELISA versus CLIA) were performed using the chi-square (*χ*²) test. For the main comparisons of proportions, absolute risk differences (RDs) with 95% confidence intervals (CIs) were calculated to provide estimates of effect size and precision in addition to hypothesis testing.

Differences in the distribution of indeterminate result causes (LMR versus HNV) across methods and years were also evaluated using *χ*² analysis or Fisher’s exact test.

Agreement between TB1 and TB2 antigen results among determinate samples was assessed by calculating overall percentage agreement and Cohen’s kappa (*κ*) coefficient with corresponding 95% CIs.

All statistical tests were two-sided, and a *P* value < 0.05 was considered statistically significant. Statistical analyses were performed using GraphPad Prism version 8.0.1 (GraphPad Software, San Diego, CA, USA).

## Data Availability

All de-identified data used in this study are available from the "DatasetData set QFT-Plus" file held in the Figshare public repository at the following URL https://doi.org/10.6084/m9.figshare.33855934.
